# Constituents of Music and Visual-Art Related Pleasure – A Critical Integrative Literature Review

**DOI:** 10.3389/fpsyg.2017.01218

**Published:** 2017-07-20

**Authors:** Marianne Tiihonen, Elvira Brattico, Johanna Maksimainen, Jan Wikgren, Suvi Saarikallio

**Affiliations:** ^1^Finnish Centre for Interdisciplinary Music Research, Department of Music, Art and Culture Studies, University of Jyväskylä Jyväskylä, Finland; ^2^Center for Music in the Brain, Department of Clinical Medicine, Aarhus University and The Royal Academy of Music, Aarhus/Aalborg Aarhus, Denmark; ^3^Max Planck Institute for Empirical Aesthetics, Department of Music Frankfurt, Germany; ^4^Centre for Interdisciplinary Brain Research, Department of Psychology, University of Jyväskylä Jyväskylä, Finland

**Keywords:** music, visual-art, pleasure, reward, enjoyment, aesthetic experience

## Abstract

The present literature review investigated how pleasure induced by music and visual-art has been conceptually understood in empirical research over the past 20 years. After an initial selection of abstracts from seven databases (keywords: pleasure, reward, enjoyment, and hedonic), twenty music and eleven visual-art papers were systematically compared. The following questions were addressed: (1) What is the role of the keyword in the research question? (2) Is pleasure considered a result of variation in the perceiver’s internal or external attributes? (3) What are the most commonly employed methods and main variables in empirical settings? Based on these questions, our critical integrative analysis aimed to identify which themes and processes emerged as key features for conceptualizing art-induced pleasure. The results demonstrated great variance in how pleasure has been approached: In the music studies pleasure was often a clear object of investigation, whereas in the visual-art studies the term was often embedded into the context of an aesthetic experience, or used otherwise in a descriptive, indirect sense. Music studies often targeted different emotions, their intensity or anhedonia. Biographical and background variables and personality traits of the perceiver were often measured. Next to behavioral methods, a common method was brain imaging which often targeted the reward circuitry of the brain in response to music. Visual-art pleasure was also frequently addressed using brain imaging methods, but the research focused on sensory cortices rather than the reward circuit alone. Compared with music research, visual-art research investigated more frequently pleasure in relation to conscious, cognitive processing, where the variations of stimulus features and the changing of viewing modes were regarded as explanatory factors of the derived experience. Despite valence being frequently applied in both domains, we conclude, that in empirical music research pleasure seems to be part of core affect and hedonic tone modulated by stable personality variables, whereas in visual-art research pleasure is a result of the so called conceptual act depending on a chosen strategy to approach art. We encourage an integration of music and visual-art into to a multi-modal framework to promote a more versatile understanding of pleasure in response to aesthetic artifacts.

## Introduction

When considering human behavior in general, striving for pleasure and reward seems to be an integral part of human behavior and a driving force in humans and in animals (Kringelbach and Berridge, 2010a,b). Indeed, pleasure, including positive and negative affect, is related to processes crucial for survival and adaptive functions; it is involved in the regulation of procreation, food intake and motivation, also it is considered a core affect in some of the main emotion models (Russel, 1980; Ledoux, 2000; Barrett, 2006; Nesse, 2012). Thus, it seems that we continuously evaluate the sensory input from our environment according to our internal states of needs and desires (Cabanac, 1971). Regarding types of pleasure, Berridge and Kringelbach (2008) separated basic pleasures (sensory and social) from those of higher-order (monetary, artistic, altruistic, musical, and transcendent), considering arts in general as higher-order pleasures. Yet, it has also been suggested that music and visual-art are not restricted to that of the higher-order pleasure. Brattico and Pearce (2013) advocated for a distinction between *immediate sensory pleasure* and *reflective process of enjoyment* in regard to music. It also seems to be common to many models of visual-art to integrate low-level feature analysis relying on the visual sensory system (bottom-up) and higher-order ways to give meanings to artworks (top-down) when aiming to explain experiences derived from visual-art (Pelowski et al., 2016). Indeed, already in Fechner’s (1876) “Vorschule der Ästhetik” the research of visual and auditory elements was under the same label of “aesthetics from below,” where the bottom-up mechanisms of music and visual-art were considered eventually to explain the top-down mechanisms of art enjoyment in general. Since then the scientific take on the influence of music and visual-art has not only become broader, causing the fields to split into several smaller sub-disciplines, but the empirical research on music and on visual-art has grown apart, and the term aesthetic experience seems to be more characteristic to the research on objects and artifacts perceived visually (Hargreaves and North, 2010; Brattico et al., 2013; Hodges, 2016). Despite overlapping research questions, and the assumption that the same components (perception, production, response and interaction) govern both, pleasure derived from music and visual-art, and art appreciation in general (Chatterjee, 2011; Bullot and Reber, 2013), empirical research of visual-art and music has had relatively little dialog with each other in the recent years.

Considering the omnipresent audio-visual culture, we live in, we acknowledge that most likely also aesthetic objects, such as music and visual-art, are likely to be integrated into our lives in an interactive loop involving the environment and the derived pleasure and emotions. As already mentioned above, positive and negative affect are known to have adaptive functions, and positive affect in particular has consequences in daily life for planning and constructing cognitive and emotional resources (Lyubomirsky et al., 2005; Fredrickson et al., 2008). The purpose of this review is not only to contribute to the unification of the two fields, but also to provide a better starting point for the growing research investigating how music and visual-art in general impact the everyday life, such as enhancement of living environments and well-being. In addition, research on pleasure and reward is a valuable contribution in affective neuroscience when doing research on affect-based psychopathologies such as eating disorders, obsession, depression and drug addiction (Berridge, 2003). We believe that research on art-induced pleasure has a position in the endeavor of elucidating the psychological constituents behind the human behavior underlying pleasure. Yet, we do not want to advocate solely for a naturalistic approach, according to which the appraisal of art objects does not need to be separated from that of any other object (Brown et al., 2011). A recent paper discussing the past and the future of neuroaesthetics, recognized three different emphases in the cognitive science of aesthetic experiences: cognitive neuroscience of aesthetics, cognitive neuroscience of art and cognitive neuroscience of beauty (Pearce et al., 2016). Following this categorisation, we focus on the neuroscience of art in general, and suggest an approach to sensory multimodality through the concept of pleasure which we consider suitable for two reasons. First, it is expected that when focusing on the term pleasure, studies dealing with the cognitive and the emotional aspects of music and visual-art engagement will be reached. Recent literature on reward demonstrate that pleasure is a much more complex phenomenon than mere hedonic response, both on the conceptual and on the functional levels (Kringelbach and Berridge, 2009; Leknes and Tracey, 2010; Smith et al., 2010). Indeed, reward seems to be constructed of different psychological components which have been characterized as affect, motivation and learning, which can further be delineated into comprising elements of affective and cognitive processes, such as wanting based on cognitive incentives and incentive salience, learning based on cognitive and associative learning, and affect consisting of explicit feelings and implicit affective reactions (Berridge, 2003). Second, it is expected that studies focusing on affective experiences, other than those of intense aesthetic experience or peak emotions, will also be captured. A qualitative thematic analysis was chosen to approach the research question in order to recognize patterns, similarities and differences in the chosen aspects of the data. The goal of this review is to understand how pleasure has been conceptualized, either directly or indirectly, in recent empirical research on music and visual-art, to eventually enable the emergence of cognitive neuroscience of art.

Here we applied the keywords of “pleasure,” “reward,” “enjoyment” and “hedonic” to evaluate how empirical music and visual-art research have approached pleasure empirically. The focus was set on the selected methods and variables, yet due to the large variability of the roles of the keywords in each paper, their positioning in the context of the research questions were investigated in further detail. Regarding the focus – pleasure – of this review, we are aware of the terminological importance of preference, expertise, beauty, liking and valence in the fields of visual-art research and music psychology (Silvia, 2008; Rentfrow and Mcdonald, 2010; Ishizu and Zeki, 2011). Yet, those terms were not included as keywords because they were considered either as too specific, or too controversial to be paralleled with pleasure (see, e.g., Bundgaard, 2015; Pearce et al., 2016). Indeed, we chose the keywords to reflect the universality of pleasure, without being too much rooted into either of the disciplines, such as beauty is rooted in neuroaesthetics, where it is used to describe the feelings an aesthetic experience can evoke, and also the perceptual features of an aesthetic object (Bundgaard, 2015). Also, liking was seen here more or less as a synonym for preference, which is often in music studies related to genre specific studies dealing with background variables, such as self-esteem, age, sex and socio-economic variables, not necessarily related to the experiential features of enjoyment (North, 2010; Corrigall and Schellenberg, 2015). Also, valence is an extremely frequently used standardized measure applied in many psychological studies. Had valence been included, it is to be expected that the focus of the review would shifted away from the experiential pleasure resulting in a very large amount of papers, exceeding the scope of the review. Also, for the sake of clarity, we aimed to define this review terminologically by focusing on music and visual-art as objects of empirical research, instead of aesthetics, or aesthetic experiences in general. Indeed, the research on visual-art is closely related to aesthetics, yet aesthetics as such comprised of a multi-disciplinary field of research and is, as a concept, not well defined and thus remains outside the scope of this review (Carroll, 2000). Because the history of empirical studies on music and visual-art is long and characterized by different research trends and emphasis (Hargreaves and North, 2010), we decided to limit the scope of the review to the recent 20 years.

## Materials and Methods

### Literature Search and Selection

**FIGURE 1:**
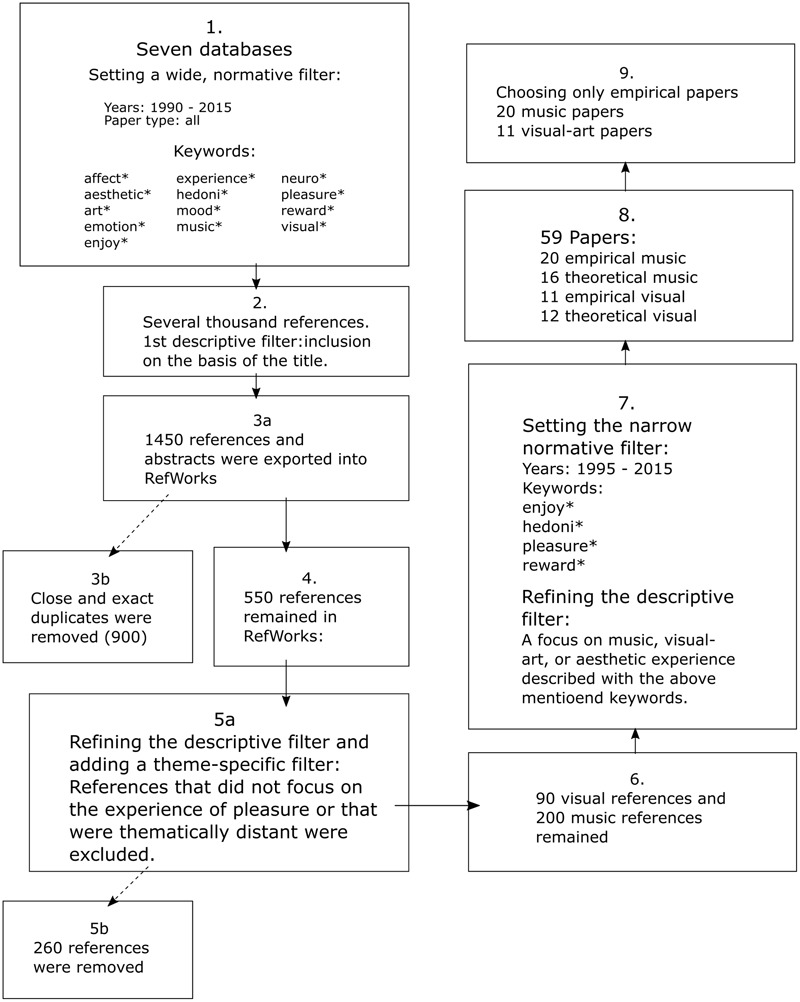
Literature search. Flowchart of the process of the literature search and selection.

The following databases were searched for literature: APA, Jstor, PubMed, Science Direct, Scopus, Web of Science, and Nelli. We followed a procedure illustrated and described below (**Figure 1**). For a more detailed walk-through, please see the Appendix I. The literature search consisted of several steps of inclusion and exclusion, and it consisted of systematically developing different types of filters while searching the literature. Also, searches were conducted by using an asterisk (e.g., pleasur^∗^, instead of pleasure) to not to oversee papers with language-based variability in the use of the key-words. The purpose of this strategy was firstly, to have an overview of the literature of both fields of interest, and secondly, to avoid losing relevant literature or overlooking crucial terminology. The first applied filter we call the normative filter, indicating that all papers which fulfilled the criteria of the wide set of keywords were searched. Thus, the data sampling strategy was comprehensive and included all the fields provided by each database search engine.

Conclusively, reports on empirical studies that focused on pleasurable, hedonic, enjoyable or rewarding experience of music or visual-art were included. Studies were also included if any of these pleasure-synonymous concepts were embedded in the context of an aesthetic experience. This resulted in 59 theoretical and empirical papers. Of these 59 references, only papers reporting on empirical studies were included, resulting in 20 music and 11 visual-art papers. For the sake of readability, hereafter the term “pleasure” is used to refer to the other keywords of enjoyment, hedonic, and reward as well.

### Data Extraction

The same core information from each paper was extracted and tabulated into a spreadsheet consisting of general publication background data (author names, journal name, year of publication, sample characteristics) and specific data extracted to answer the research questions. As far as possible, the data were copied directly as they were stated in the corresponding article, and the tabulated data were then used as a source for drawing further conclusions and categorizations for the subsequent synthesis and analysis.

### Data Synthesis

Here, the synthesis was conducted mostly in a narrative form to identify patterns in the data, and to strive for a more holistic understanding of the conceptualization of pleasure (Rumrill and Fitzgerald, 2001), yet in order to support the findings the tabulated aspects were also quantified. Since this review aims to understand how pleasure has been approach in empirical research, it was decided to focus on inspecting the taken methodologies and variables. Despite the systematic appliance of the filters, while searching the literature, a great variance among the papers regarding the keywords could still be detected. It is due to this reason that the role of the keyword and the type of the research question were further categorized. Thus, the decision to focus the synthesis on the two other aspects – role of the keyword and type of the research question – emerged from the included papers, that is, they were not predetermined.

#### The Role of the Keyword in Relation to the Research Question

The papers were first categorized according to two different positions of the keyword, either as direct or indirect. If the role of the keyword was considered direct, the keyword was clearly the object of inquiry. Whereas, if the role of the keyword was not the main target of the inquiry but, rather, an attribute of the main object of research, it was considered to be indirect. Here it should be noted that because the term aesthetic experience was included, the role of the keyword was considered indirect if it was used in that context (e.g., Belke et al., 2010 where the main term is aesthetic or art appreciation, yet it is constantly described with terms of hedonic or pleasure).

#### Type of Research Question

The types of research questions were divided into three categories: External factor-driven, internal factor-driven, and impact-driven. The studies in the first category posed questions in which external factors were considered to influence the internal state of the perceiver (e.g., how musical expressivity influences the derived pleasure). In the second category, the experience was investigated from the perspective of the subject (e.g., the experience depended on the perceiver’s personality). Finally, some studies used the experience of pleasure to investigate other phenomena, and these questions were labeled as impact-driven questions (e.g., the influence of music-induced pleasure on learning outcomes). The questions were categorized on the basis of how the research question was postulated in the corresponding paper without consideration of single variables of the experimental setting. Because many papers had several questions, the question could be categorized under two types, both internal- and external-driven questions. Therefore, more than one type of research question was tabulated for each paper.

#### Methods and Main Variables

The methods applied in each paper were tabulated according to the following criteria: “Neuroimaging” refers to methods of brain imaging and brain neurophysiology. “Behavioral” refers to tasks given to the participants, usually consisting of music listening or picture viewing, and the subsequent rating of the stimuli. “Questionnaire, Interview” refers to studies using online or pen and paper questionnaires or interviews. “Physiological measures” refers to objective, psychophysiological measurements such as heart rate. Only a maximum of two methods were tabulated for each paper.

Additionally, the main variables of each study were tabulated to obtain more detailed information on the variables measured. Because most studies used a large variety of different variables, only the most frequently used ones were categorized and discussed in relation to the enlisted methods (see Appendix II).

### Analysis

In the analysis, we aim to identify aspects of music and visual-art-induced pleasure that are missing, incomplete, or poorly represented in the literature (Torraco, 2005). The tabulated results are inspected as an entirety on the experiential level in reference to stimulus features, perceiver attributes, cognitive-perceptual, and emotional attributes. Finally, the results are discussed in the light of pleasure conceptualisation in the interdisciplinary literature of philosophy and affective neuroscience as introduced in the beginning of the review. Further it is also discussed, whether pleasure is learned or instinctual, biological or cultural, universal or individual, and whether pleasure is a result of action or whether it facilitates the pursuit of actions (Sizer, 2013; Matthen, 2017).

## Results

Altogether 20 papers were found in the music domain, and 11 papers in the visual-art domain. In both fields, the majority of the papers were published after the year 2008. The extracted information is tabulated below. In the **Table 1** the role of the keyword (direct or indirect) is assigned to the corresponding field of either music or visual-art. In the **Table 2** the applied methods (brain physiology, questionnaire and interview, behavioral and psychophysiology) are cross-tabulated with the questions types (external, internal, impact or external and internal) for each domain.

**Table 1:** Tabulation of the results based on the role of the key word.

Role of the keyword	Music	Visual-art
Direct	17	2
Indirect	3	9

**Table 2:** Cross-tabulation of the results based on the role of the key word, question type and applied research methods.

Question type	Total		Brain physiology		Questionnaire and Interview		Behavioral		Psychophysiology	
External	M5	V1	M3	V1	M1	V0	M1	V0	M3	V1
Internal	M8	V4	M2	V2	M3	V1	M4	V0	M5	V3
Impact	M3	V0	M0	V0	M3	V0	M0	V0	M2	V0
Extr. and Intr.	M4	V6	M1	V3	M3	V2	M0	V1	M1	V5

*The letter M preceding the numbers refers to music, and the letter V to visual-art.*

### Role of the Keyword

As **Table 1** shows, the majority of the music papers had pleasure as a clear object of investigation. Examples of pleasure clearly being the object of investigation were, e.g., musical reward responses, music reward experiences, and reward circuitry of the brain (Montag et al., 2011; Mas-Herrero et al., 2013, 2014). Among the music papers, only in three studies the role of the keyword was said to be indirect. The rewarding aspects of music-evoked sadness, emotional rewards of music, and reward-related activation are examples of the indirect use of keywords (Zentner et al., 2008; Chapin et al., 2010; Taruffi and Koelsch, 2014).

Because the term aesthetic experience was used frequently in the visual-art papers, the keyword was often embedded in the aesthetic context. The keyword had a direct role in a minority of the papers. The indirect keywords were used to describe concepts such as aesthetic pleasure, aesthetic experience, beauty, pictorial perception and aesthetic appreciation. In addition, the terms *aesthetic experience* and *pleasure* or *aesthetic pleasure* were occasionally used interchangeably. In one of the two articles in which the keywords could be said to also be the objects, the focus was on intrinsic reward manifested in neural correlates (Lacey et al., 2011). The second article focused on the so-called hedonic principle, which was considered to be the underlying mechanism of motivation to spend a certain amount of time viewing pictures (Kron et al., 2014).

A clear difference between music and visual-art papers was the use of the actual keywords. Sixteen of the 20 music papers included reward- and/or pleasure-related terminology, whereas hedonic and enjoyment-related terms were a clear minority, used in only four of 20 articles. In regard to the keywords of the visual-art papers, the terms pleasure and hedonic were the most frequently used terms, whereas the term reward played a central role in only one of the studies, in which it also was the object of the research (Lacey et al., 2011).

### Question Type

**Table 2** illustrates the findings related to the type of question asked in the examined literature. Most external factor-driven papers (five music papers and one visual-art paper) investigated neural correlates or neural mechanisms underlying pleasure. For example, the aim was to test whether limbic and paralimbic brain areas were activated during passive music listening when participants were not given an explicit instruction to focus on emotions (Brown et al., 2004); or to map out neural mechanisms underlying mildly and intensely pleasurable music (Blood and Zatorre, 2001). The visual-art study sought to determine whether the activation of the reward circuitry took place solely from the process of recognizing that an image is artistic rather than non-artistic in nature (Lacey et al., 2011). The remainder of the external factor-driven questions aimed to recognize the quality and frequency of the reported emotions, and how these emotions could be categorized (Zentner et al., 2008; Taruffi and Koelsch, 2014) or whether liking depended on the order in which the stimuli were heard (Parker et al., 2008).

In the internal factor-driven music papers, the variables that depended on the perceiver’s attributes were arousal, familiarity, anticipation, musical knowledge and, most of all, personality traits. For example, research investigated individual variation in the experience of reward caused by money or music (Mas-Herrero et al., 2013, 2014); or whether familiarity and arousal correlated with pleasure (van den Bosch et al., 2013). Among the visual-art papers, one of the studies implementing an internal factor-driven approach tested whether the process of perception (ambiguous vs. non-ambiguous portraits) itself depended on the aesthetic experience of the viewer (Boccia et al., 2015). The second paper investigated whether emotions influenced aesthetic experience (Marković, 2010).

Visual-art papers typically included both question types. The experiments were designed to test several different variables according to the stimulus features, the perceiver, and their correlation. The relationship between the internal and external factors was thematised in several research questions. For example, a study conducted by Cupchik et al. (2009) aimed to investigate, on one hand, how different modes of viewing (aesthetic vs. pragmatic viewing mode) paintings influenced the experience and, on the other hand, how the experience depended on the structural (soft edges vs. hard edges) content of the paintings. The impact-driven questions of the music papers addressed learning, stress, attitude and music information seeking and how these factors were related to pleasure (e.g., Gold et al., 2013; Perlovsky et al., 2013).

### Methods and Main Variables

Both fields used functional magnetic resonance imaging (fMRI) most frequently (e.g., Menon and Levitin, 2005; Montag et al., 2011; Jacobs et al., 2012; Boccia et al., 2015). Also, it is notable that in music studies it was common to apply questionnaires and interviews, and physiological measures, whereas these were a clear methodological minority in the visual-art papers. However, as visible from the cross-tabulation of **Table 2**, most studies applied more than one method, which is why comparing the different methods is hard and the subsequent discussion is more interesting when considering the taken variables as well (see Appendix II for more details). In the following, we aim to provide a characterisation of the common combinations of variables and methods typical in both fields of interest.

The main variables of the imaging methods common to the music papers were the neural correlates of reward and intense pleasure or liking (e.g., Blood and Zatorre, 2001; Menon and Levitin, 2005; Montag et al., 2011; Salimpoor et al., 2011), whereas the visual-art papers addressed the difference between basic visual processing and aesthetic emotional processing, hence imagining the brain more broadly focusing on brain areas involved in pictorial processing (e.g., Jacobs et al., 2012; Kreplin and Fairclough, 2013). One of the visual-art studies addressed a question similar to those addressed in the music studies: whether the artistic status of a picture alone can activate the reward center in the brain (Lacey et al., 2011). The variables of the studies combining imaging and viewing and rating-based behavioral tasks varied including naturalness, beauty and roughness; valence and complexity; liking; classification between artistic and non-artistic statuses; aesthetic preference; reaction time or familiarity, demonstrating that in addition to perception modes, the influence of stimulus features was measured.

The most common variable in the music studies was valence, including its different variations from liking to disliking or from pleasing to not pleasing (a total of 12 studies: e.g., Parker et al., 2008; Salimpoor et al., 2011). Arousal was also frequently measured (in a total of eight studies) (e.g., Salimpoor et al., 2011; Mas-Herrero et al., 2014). In visual-art studies, valence or a similar dimension was measured in five studies (e.g., Vessel et al., 2012; Kron et al., 2014). In addition to mere liking or enjoyment, visual-art studies implemented more complex measures such as beauty, endorsement, aesthetic preference, and emotional movement (e.g., Lacey et al., 2011; Hager et al., 2012; Jacobs et al., 2012; Vessel et al., 2012). Arousal was measured in only one study (Kron et al., 2014). The music studies used character inventories, such as Behavioral Inhibition/Behavioral Approach System (BIS/BAS) or Temperament and Character Inventory (TCI), to mention a few (Montag et al., 2011; Mas-Herrero et al., 2013), and questionnaires to address the listening background or music preference (Garrido and Schubert, 2011; Gold et al., 2013). In visual-art papers, frequently addressed modes or judgmental aspects were artistic vs. non-artistic, pragmatic vs. aesthetic, emotional introspection vs. external object identification and evaluative vs. emotional components.

In sum, a common method used in both fields was brain imaging. Furthermore, when the object of research was reward or pleasure, the object was mainly thought to consist of self-reports based on valence and on psychophysiological measurements (in music studies) or different modes of judgment or perception (in visual-art studies), which had neural correlates as their reference. In the visual-art field, subjective perception was highlighted without additional objective measures. This approach was used to investigate the degree to which pleasure or the aesthetic experience depended on varying modes of perception. Thus, the subjective preparedness and focus of attention were considered the starting points for the whole experience. Music studies used both subjective and objective measures: The conscious, subjective valence and the objectively quantified parameters – such as activation of the reward circuitry or psychophysiological parameters – were required for an experience to be considered pleasurable or rewarding. Few studies aimed to test whether the stimuli used could activate reward-related brain circuitry without conscious listening or viewing.

Valence and related measures were variables that were commonly examined in both fields. Visual-art studies additionally used complex experiential and stimulus-derived descriptors, whereas the music studies collected person-derived data on background, personality and music consumption. In music studies, the more frequent use of psychophysiological measures indicates that arousal was addressed more often. In the visual-art studies, pictures of paintings, drawings or photos were used as stimuli. In all studies, the stimuli were selected by the experimenter, and many studies mixed abstract and representational stimuli. Also, one production task was given where the participants were instructed to depict affectively expressive content (Takahashi, 1995). In contrast, in the music studies, the frequent use of different questionnaires revealed the lack of real-time music stimuli, since these studies relied on retrospective memory retrieval and on participants’ conception of their own identity as music consumers: typically, these studies aimed at developing an instrument or at identifying induced emotions. One questionnaire study implemented music listening as part of the data collection (Vuoskoski and Eerola, 2011). With two exceptions (Blood and Zatorre, 2001; Montag et al., 2011), all music stimuli were pre-selected, either by a separate group of participants or by the experimenters.

## Discussion

**FIGURE 2:**
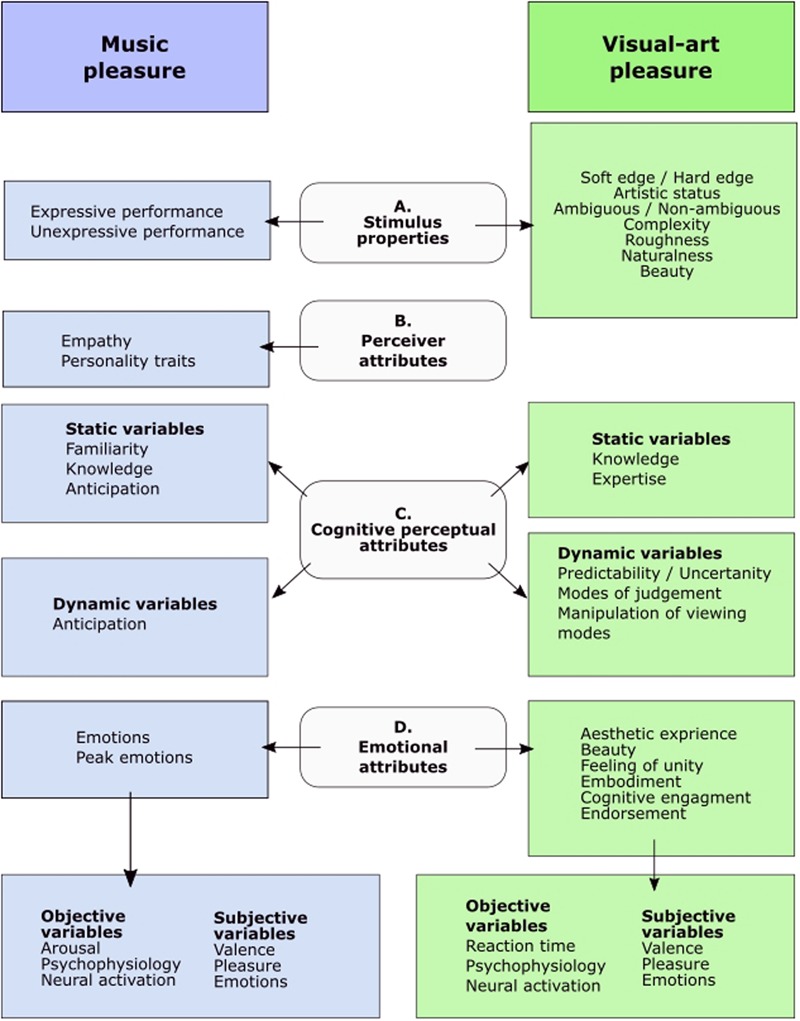
A summary of the findings. Diagram of the distribution of the most frequently examined variables in empirical research on visual-art and music-induced pleasure based on the review of the literature.

Overall, the reviewed papers demonstrate a great variety in the ways in which music and visual-art papers address pleasure. The **Figure 2** below was constructed to illustrate and structure the results in regard to stimulus properties (A), perceiver attributes (B), cognitive perceptual attributes (C), and emotional attributes (D). The **Figure 2** was constructed around the above-mentioned features to open the results of the review in the experiential context. Thus, rather than further discussing the experimental settings such as variables and methods, with the **Figure 2**, we hope to synthesize the most prominent features characterizing the experience of listening to music or viewing art, prevalent in both domains. This way we wish to lead the discussion to the more in-depth analysis of the results. Each of the above-mentioned aspects of the examined literature is discussed below. Please, see the Appendix II for the detailed tabulation of the data.

(A) **Stimulus Properties**
  Stimulus properties refer to different audible or visible qualities of music and visual-art. Here the comparison showed that in visual-art research, the role of the stimulus was emphasized in a very versatile manner. By contrast, music research emphasized the perceiver’s personal background and biographical factors, which are visible when inspecting the perceiver attributes (B), and cognitive perceptual attributes (C).

(B) **Perceiver attributes**
  Perceiver attributes refer to the individual and biographical qualities of the perceiver. Only the music research addressed listener attributes using various types of character inventories and collected data on biographical information.

(C) **Cognitive perceptual attributes**
  This level refers to the cognitive process of perceiving the stimulus. Here, instead of comparing the two fields in regard to the methods and their variables, we aimed to summarize the results by categorizing the variables in regard to the very fundamental differences among music and visual-art. Namely, music evolving in time and visual-art being static, and spatially distributed. Static variables refer to variables that accumulate over time (e.g., as a result of learning), are more biographical, and are relatively unchangeable features of the perceiver. Dynamic variables refer to attributes that can be consciously manipulated (as in visual-art research, e.g., viewing mode) or that strongly depend on the corresponding stimulus (e.g., anticipation based on the temporal evolvement of a certain musical piece). Indeed, in the field of visual-art, the range of dynamic variables is much larger, giving the perceiver an active role as an interpreting subject. Thus, it seems that whereas music evolves in time, the applied measures are static, and visual-art which is spatially distributed and temporally static, is investigated more by using variables prone to change and conscious manipulation. This approach, in which the person categorizes and actively interprets information, has also been recognized in emotion research, for example, by Barrett (2006). She called this the “conceptual act” (as opposed to emotions as “natural kind entities”). Specifically, she stated that emotions emerge as a result of people applying their previously acquired knowledge to process and categorize sensory information. Conclusively, many experimental set-ups relied on the perceiver’s ability to vary the mode of viewing art and recorded whether this changed the resulting experience. Instead of highlighting personality traits or general background, such research considered the viewer as an active participant in the experience through his or her perceptual and interpretational input during the actual viewing situation. By implementing these various modes of perception, and by changing the stimulus features, scholars often attempted to capture the degree to which the derived experience depended on the judgmental or experiential/emotional mode.

(D) **Emotional attributes**
  Here, it becomes evident that both fields addressed emotional dimensions of an experience by applying subjective and objective measures. Research conducted in the field of music focused generally on emotions – including also negative emotions – whereas visual-art research often approached pleasurable experience by using rather complex, abstract, and evaluative terminology such as endorsement and being moved. To approach the different types of emotions and experiences, both fields measured the degree of experienced valence. Valence and arousal are dimensions that are commonly applied in emotion psychology to characterize different emotional qualities. For example, Feldman Barrett and Russell (1999) postulated that valence and arousal are independent of each other, and that both have independent polarities. Indeed, it in the visual-art field, arousal was not commonly used as a dimension of a pleasurable experience. This was also evident in the lack of physiological measurements, which are typically applied to measure arousal. In the music studies the applied arousal measures were usually objective psychophysiological measurements, even though arousal can also be applied, e.g., in the form of questionnaires as a subjective self-report.

In music papers, a typical underlying conceptualization was intrinsic reward, which is discussed as a dimension in appraisal theories. Intrinsic pleasantness represents a rather early reaction in the unfolding chain of events of appraisal, and it is considered to determine the fundamental reaction to an already detected stimulus encouraging avoidance or approach (Ellsworth and Scherer, 2003). First, many papers aimed to demonstrate that music is indeed intrinsically rewarding. Second, the interaction between cortical and subcortical brain regions was investigated to elaborate how one derives pleasure from abstract sounds.

In summary both fields do represent in the philosophical literature of sensory affect prevalent anti-representational view, in that they separate the experience from the objective features of the stimulus, such that the locus of affect is indeed the experience of the individual, and that the phenomenology of the sensation is not explained by the stimulus features (Aydede and Fulkerson, forthcoming). In the visual-art field the conceptualisation of sensory affect can be inspected in the light of attitudinal or externalist theories. In accordance to these theories, the pleasant sensation to the sensory features of the stimulus, together with a mental attitude – such as desiring, wanting, preferring and liking – construct the composite state of a pleasurable sensation. Crucial here is the idea that sensory pleasure is strongly connected to mental states without having an intrinsic qualia, and thus it is causally connected to the current state of the person (Aydede and Fulkerson, forthcoming). In visual-art the explanatory power to the differences in the experience is given to knowledge, intentionality, history and time. According to an imperative view in the philosophy of sensory affect, sensory information presents in itself command-like information to the organism, which informs the organism to action or to retain from an action. Thus, sensory information are considered as motivational states (Aydede and Fulkerson, forthcoming). This kind understanding of pleasure seems prevalent in papers which address the stimulation of the reward center of the brain. Yet an approach more refined and closer to the understanding of affective neuroscience seems to be the psychofunctionalist view, according to which incoming sensory information is valued in causal and functional roles such that the information still holds motivational components, yet it is integrated to the mental economy of the perceiver.

## Conclusion

This literature review aimed to understand how pleasure derived from music and visual-art had been understood conceptually, either directly or indirectly, in empirical research during the past 20 years. The papers were analyzed in qualitative terms, instead of a quantitative meta-analysis, due to the small amount of papers and due to the large variability in the operationalisation of the key words. The distinction between direct and indirect keyword use is a good example of qualitative comparison, where the papers being reviewed guide the question formulation, which might mean that the formulation of the research question can change during the review process. It turned out that in particular in the visual-art papers pleasure was a very vaguely used term that is, many times it was not a clear object of investigation but rather, it was a characterisation of the researched phenomenon. In our view, an informative quantitative meta-analysis would have required more common nominators and less divergence among the papers. The first findings emerged already during the literature search that, after having applied descriptive, theme-specific and normative filters, started from approximately 200 papers in music, and 90 papers in visual-art and, after refining the keywords and filters, ended up with 20 in music and 11 visual-art studies. The clearly smaller amount of visual-art papers in comparison to the music papers, is a clear demonstration that the phenomenon of interest – pleasure – had a different position in visual-art research. This is also highlighted by the fact that the keywords in the visual-art papers were frequently embedded into the context of an aesthetic experience. Yet, as demonstrated in the literature search flowchart, the ratio between the fields was more balanced when the theoretical papers were also included. This is an indication that pleasure has a more concise role in theories and models of visual-art than in the equivalent empirical research.

Next to the literature search, the actual synthesis confirmed the above-discussed findings. Music and visual-art studies showed an emphasis on different keywords (reward and pleasure in music research, hedonic and pleasure – embedded in aesthetic experience – in visual-art) and appointed different roles for the keywords (more direct in music, indirect in visual-art), thus demonstrating that pleasure is not a scientifically unanimously defined, nor a conceptually clear object of investigation. Indeed, the process of choosing the correct keywords was a result of several discussions, thus also highlighting the definitional issues related, on one hand to the phenomenon of interest, and on the other hand, on the differences between the two fields. The focus of this review was not aesthetic experiences as such, yet had we included beauty as a keyword, and had papers solely focusing on aesthetic experience, without a clear connection to pleasure, also been included, then the balance between the papers would have been different. Whereas the term aesthetic experience is prevalent in the field of visual-art, a similarly important term in the field of music is the term “peak emotion” or “strong emotion” which often investigate the psychophysiological chills, also known as goose pimples (See, e.g., Gabrielsson, 2010; Grewe et al., 2011). Nevertheless, chills, nor the specific terminology related to the peak emotions were included as keywords because they, too, would have been too specific compared with the more general terms related to pleasure. Also, characteristic to chills is that they may occur in response to unpleasant events, which would have stretched the scope of the review. We assume that the reason why the concept of pleasure seems to play a larger and a more direct role in the empirical music research than in the empirical visual-art research lies in the different backgrounds of the disciplines. The prevalence of the term “reward” in the music studies can probably be traced down to the field of affective neuroscience, where it typically refers to the activation of the reward circuitry of the brain and is concerned with mapping the neural basis of mood and emotional processing of the brain (Dalgleish, 2004). The history of empirical research on music and visual-art is long, yet the scope of the review was short, comprising the past 20 years of research to only include relatively recent literature. During this time the term neuroaesthetics was coined (Ishizu and Zeki, 2011) (see also Zeki, 1999), which is a sub-discipline of cognitive neuroscience, focusing on understanding how the brain processes pictorial information and beauty, and which biological functions underlie these processes; the degree to which a good pictorial organization underlies aesthetic experiencing; and how an aesthetic experience becomes a conscious one (Di Dio and Vittorio, 2009; Chatterjee, 2011). Indeed, rather than searching for the correlates in the reward center of the brain, neuroaesthetics has been more concerned with finding common nominators among the stimuli which are artistically appreciated and liked (Ramachandran and Hirstein, 1999), thus possibly explaining the difference in the use of the keywords. In contrast, the background of the music papers lies in emotion psychology, which most likely explains why pleasure was often discussed and investigated in emotion related terms. The fact the music studies did not address the variation of the stimulus features in similar scale as the visual-art is surprizing, considering the fact the question about the link between musical features and the corresponding emotions has been a traditional topic in music psychology. Yet one of the fundamental differences between the art forms is the fact that they employ different sensory systems and also, they are culturally integrated in our daily lives in a different manner. This difference might lie in the cultural significance of our visual perception as our dominant sense and that we are most accustomed to extracting semantic meaning from and ascribing it to visual representations.

We can conclude that music research conceptualized pleasure by using elements of core affect or hedonic tone (valence and arousal) (Feldman Barrett and Russell, 1999; Russell and Barrett, 1999) and intrinsic reward. In particular, the idea of music being able to activate the reward center and the use of psychophysiological measures refer to the idea of music-induced pleasure being biological, rather than culture and context specific in nature. It seems, as if musical pleasure was more involved in the homeostasis of the organism, having an access to the parts of the nervous system which are not subjected to volitional control of the person such as autonomic nervous system and limbic structures of the brain. This aspect is also highlighted when inspecting musical pleasure in terms of the survival circuits and functions related to that, such as motivation, emotions, reinforcement and arousal (LeDoux, 2012). Although an element of core affect – valence – was also common in visual-art research, the derived pleasure was considered to emerge as a result of the conceptual act (Barrett, 2006). That is, the experience is dependent on the perceiver’s active interpretation and attribution of meaning, referring to a more culture and context specific understanding of pleasure (see, e.g., Bullot and Reber, 2013). It seems that visual-art pleasure was conceptualized more as an act of information processing consisting of the duality of feature processing and representation (Marr, 1982). Inspecting the results on the dichotomy of learning and instinct, it seems that in both domains it was rather learning-, than instinct-based factors that were dominant. With some variance, both discussed expertise, familiarity and anticipation, which can be seen as examples of accumulative learning (Silvia, 2008; Huron, 2010). Also, both domains highlighted the importance of individuality over universality in response to the stimuli, yet different aspects were highlighted. Music research focused on subject-driven parameters such as familiarity, biographical background and personality, which seem to be rather stable features and inaccessible to voluntary modulation of the perceiver. Whereas in the field of visual-art, the experience was particularly conceived a result of a conscious, and an active process of interpretation, depending on dynamic variables subjected to the level of expertise and personal control.

As demonstrated in the beginning of this review pleasure and the human desire for pleasure facilitates mental processes and behavior. In literature on pleasure, it has been discussed whether pleasure facilitates the pursuit of an activity, or whether it is the result of an activity (Sizer, 2013; Matthen, 2014). Mainly due to the fact that pleasure had such a variant role in the papers reviewed here, no conclusion about such a relationship could be made. Yet, exactly the questions how art-induced pleasure and reward mediate human behavior and mental processes, or how different pleasure systems (Berridge and Kringelbach, 2015) underlie pleasurable experiences are particularly intriguing ones, and indeed, have been highlighted in the recent literature (Chatterjee and Vartanian, 2016; Pearce et al., 2016). Ultimately, with this review we wish to encourage future empirical research to approach pleasure and its mediating role for cognition and affect from the multimodal perspective of music and visual-art. Yet, as long as music and visual-art research are not integrated and they lack a shared framework, the research on sensory multimodality will remain difficult and restricted (Marin, 2015; Hodges, 2016). Also, we hope that future comparative research would reveal certain modality-specific characteristics in emotional responses to music and visual-art, leading to a more realistic and versatile understanding of enjoyment, not only on the conceptual, but also on the sensory level.

## Author Contributions

SS, JW, JM, and MT defined the scope of the review (keywords, inclusion- and exclusion criteria), the goal and the purpose of the paper. Additionally, JW, SS, and JM commented on the text of the paper. Also, SS is the thesis supervisor of the first author and she provided methodological support as well. EB commented on the paper, provided discussion input and was involved in the process of writing as well.

## Conflict of Interest Statement

The authors declare that the research was conducted in the absence of any commercial or financial relationships that could be construed as a potential conflict of interest.
