# Supplementary material for: Constituents of Music and Visual-Art Related Pleasure – A Critical Integrative Literature Review
Source: Front Psychol. 2017 Jul 20;8:1218. doi: 10.3389/fpsyg.2017.01218 (PMC5517436; doi:10.3389/fpsyg.2017.01218)
Supplement: Supplementary file 1 [file Data_Sheet_1.PDF]

Appendix I

Constituents of music and visual-art related pleasure - A critical integrative literature review

Tiihonen, Marianne[1], Brattico, Elvira[2], Maksimainen, Johanna[1], Wikgren, Jan[3], & Saarikallio, Suvi[2]

1 [1] Finnish Centre for interdisciplinary Music Research, Department of Music, University of Jyväskylä, Jyväskylä, Finland

2 Centre for Music in the Brain (MIB), Department of Clinical Medicine, Aarhus University & The Royal Academy of Music Aarhus/Aalborg, Aarhus, Denmark

3 Centre for Interdisciplinary Brain Research, Department of Psychology, University of Jyväskylä, Jyväskylä, Finland.

1.0 Literature Search

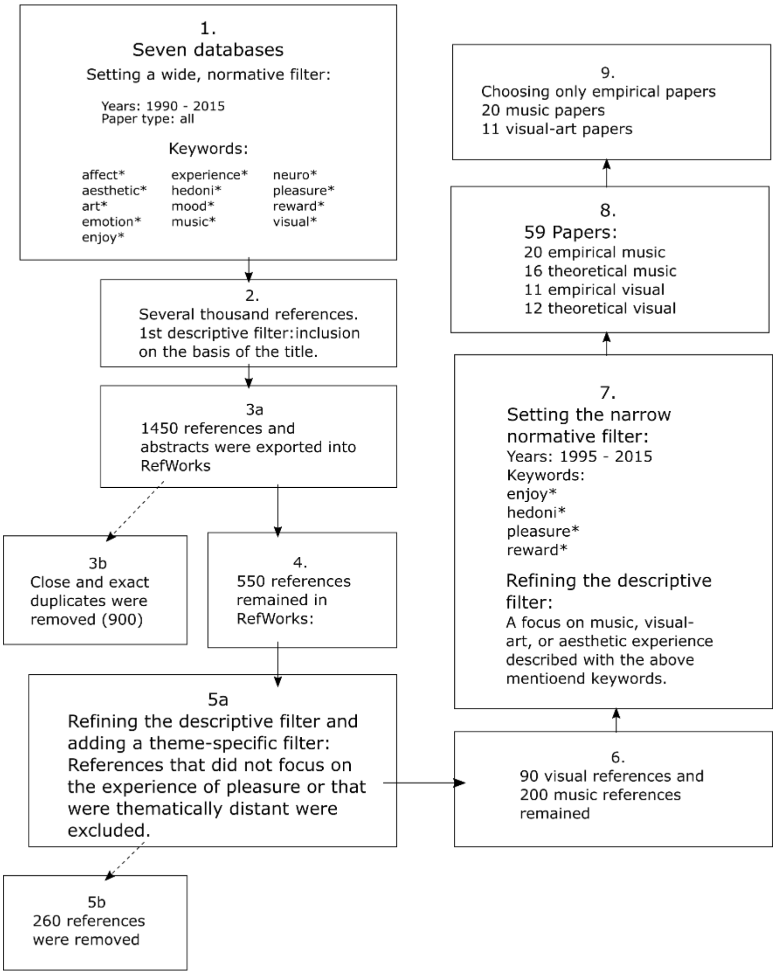

Figure 1. Flowchart of the literature search

1. The following databases were searched: APA, Jstor, PubMed, Science Direct, Scopus, Web of Science, and Nelli. First a wide normative filter was applied. This means that all paper types between the years 1990 and 2015 were included, also the set of used keywords was wide: affect, aesthetic, art, emtion, enjoy, expereince, hedoni, mood, music, pleasure, reward and visual. The purpose of applying a wide filter was to receive an overview of the literature about music, visual-art, and aesthetics.

2. The first inclusion was based on the descriptive filter: The references, which, based on their title, broadly dealt with a topic related to experiential or perceptual dimensions of visual-art, music and aesthetics were exported to the reference management tool RefWorks.

3 a) From all databases 1450 references were exported into RefWorks.

3 b) Close and exact duplicates were removed using RefWork´s search. 900 references were removed.

4. After removing duplicates, 550 references remained in RefWorks for further inspection.

5 a) The references of the remaining papers were read and inspected carefully to set further filters. For example, here the dominance of the term "aesthetic experience" became so evident, that the visual-art papers had to be considered in the context of the aesthetic experience, even though the aesthetic experience as such was not the focus of this review. Also, next to the already existing normative and descriptive filter, a theme-specific filter was applied. This allowed the exclusion of papers with the following distant themes and topics:

- Education related articles
- Specific artists and their interpretation
- Infancy related studies
- Music therapy
- Clinical research (music therapy, brain damage, depression etc.)
- Studies that assess only one gender
- Performance or emotion perception of third parties
- Movement/motoric related studies
- Creativity research
- Bodily aesthetics
- Drug studies
- Psychoanalytic studies
- Studies conducted with only professional musicians / art expertise studies
- Marketing studies
- Studies from a very distant discipline such as religion
- Emotional – not artistic – pictures, such as facial expressions
- Artist´s intentions or interpretations
- Studies aiming at emotional computing
- Pictorial aesthetics of music culture
- Papers explaining music or arts from an evolutional perspective
- Articles focusing on the systematic implementation of emotional self-regulation mechanisms from the perspective of the user.
- Vocabulary based investigations e.g. (concept of beauty: Which adjectives describe aesthetic beauty)
- Personality traits in combination with emotions
- Studies related to aesthetic preference
- Designer aesthetics
- Studies investigating liking and preference
- Studies concentrating on artist´s intentions
- Emotion recognition studies

Also, the following paper types were excluded:

- Essay collections
- Book Reviews
- Chapters in books

5 b) The process above resulted in removing 260 references.

6. At this stage the ratio between music and visual-art papers was approximately 2:1.

7. To further systematically narrow down the scope of the review, the normative filter applied in the initial stage of the search was narrowed by refining the keywords. Accordingly, the descriptive filter was also refined: the phenomenon of interest had to be described using at least one of the keywords defined in the normative filter. Additionally, restrictions regarding the article types were made: essay collections, book reviews and chapters in books were excluded.

8. After going through the references and applying the narrow normative and descriptive filter, 59 theoretical and empirical papers were left over.

9. Out of the 59 references only papers reporting on empirical studies were included.

2.0 Reviewed Literature

2.1. Music

Blood, A. J., & Zatorre, R. J. (2001). Intensely pleasurable responses to music correlate with activity in brain regions implicated in reward and emotion. *Proceedings of the National Academy of Sciences of the United States of America, 98*(20), 11818-11823. doi:10.1073/pnas.191355898

Brown, S., Martinez, M. J., & Parsons, L. M. (2004). Passive music listening spontaneously engages limbic and paralimbic systems. *Neuroreport, 15*(13), 2033-2037. doi:10.1097/00001756-200409150-00008

Chapin, H., Jantzen, K., Kelso, J. A., Steinberg, F., & Large, E. (2010). Dynamic emotional and neural responses to music depend on performance expression and listener experience. *PloS One, 5*(12), e13812. doi:10.1371/journal.pone.0013812

Dube, L., & Morin, S. (2001). Background music pleasure and store evaluation - intensity effects and psychological mechanisms. *Journal of Business Research, 54*(2), 107-113. doi:10.1016/S0148-2963(99)00092-2

Garrido, S., & Schubert, E. (2011). Individual differences in the enjoyment of negative emotion in music: A literature review and experiment. *Music Perception: An Interdisciplinary Journal, 28*(3), 279-296.

Jacobs, R. H. A. H., Renken, R., & Cornelissen, F. W. (2012). Neural correlates of visual aesthetics - beauty as the coalescence of stimulus and internal state. *Plos One, 7*(2) doi:10.1371/journal.pone.0031248

Laplante, A., & Downie, J. S. (2011). The utilitarian and hedonic outcomes of music information-seeking in everyday life. *Library and Information Science Research, 33*(3), 202-210. doi:10.1016/j.lisr.2010.11.002

Mas-Herrero, E., Marco-Pallares, J., Lorenzo-Seva, U., Zatorre, R. J., & Rodriguez-Fornells, A. (2013). Individual differences in music reward experiences. *Music Perception: An Interdisciplinary Journal, 31*(2), 118-138.

Mas-Herrero, E., Zatorre, R. J., Rodriguez-Fornells, A., & Marco-Pallares, J. (2014). Dissociation between musical and monetary reward responses in specific musical anhedonia. *Current Biology, 24*(6), 699-704. doi:10.1016/j.cub.2014.01.068

Menon, V., & Levitin, D. J. (2005). The rewards of music listening: Response and physiological connectivity of the mesolimbic system. *Neuroimage, 28*(1), 175-184. doi:10.1016/j.neuroimage.2005.05.053

Montag, C., Reuter, M., & Axmacher, N. (2011). How one's favorite song activates the reward circuitry of the brain: Personality matters! *Behavioural Brain Research, 225*(2), 511-514. doi:10.1016/j.bbr.2011.08.012

Parker, S., Bascom, J., Rabinovitz, B., & Zellner, D. (2008). Positive and negative hedonic contrast with musical stimuli. *Psychology of Aesthetics, Creativity, and the Arts, 2*(3), 171-174. doi:10.1037/1931-3896.2.3.171

Perlovsky, L., Cabanac, A., Bonniot-Cabanac, M., & Cabanac, M. (2013). Mozart effect, cognitive dissonance, and the pleasure of music. *Behavioural Brain Research, 244*, 9-14. doi:10.1016/j.bbr.2013.01.036

Salimpoor, V. N., Benovoy, M., Larcher, K., Dagher, A., & Zatorre, R. J. (2011). Anatomically distinct dopamine release during anticipation and experience of peak emotion to music. *Nature Neuroscience, 14*(2), 257-262. doi:10.1038/nn.2726 [doi]

Salimpoor, V. N., Benovoy, M., Longo, G., Cooperstock, J. R., & Zatorre, R. J. (2009). The rewarding aspects of music listening are related to degree of emotional arousal. *Plos One, 4*(10), e7487. doi:10.1371/journal.pone.0007487

Salimpoor, V. N., van den Bosch, I., Kovacevic, N., McIntosh, A. R., Dagher, A., & Zatorre, R. J. (2013). Interactions between the nucleus accumbens and auditory cortices predict music reward value. *Science, 340*(6129), 216-219. doi:10.1126/science.1231059

Taruffi, L., & Koelsch, S. (2014). The paradox of music-evoked sadness: An online survey. *Plos One, 9*(10), e110490. doi:10.1371/journal.pone.0110490

van den Bosch, I., Salimpoor, V. N., & Zatorre, R. J. (2013). Familiarity mediates the relationship between emotional arousal and pleasure during music listening. *Frontiers in Human Neuroscience, 7*, 534. doi:10.3389/fnhum.2013.00534

Vuoskoski, J., K., Thompson, W. F., McIlwain, D., & Eerola, T. (2012). Who enjoys listening to sad music and why* *Music Perception: An Interdisciplinary Journal, 29*(3), 311-317.

Zentner, M., Grandjean, D., & Scherer, K. R. (2008). Emotions evoked by the sound of music: Characterization, classification, and measurement. *Emotion, 8*(4), 494-521. doi:10.1037/1528-3542.8.4.494

2.2. Visual-art

Belke, B., Leder, H., Strobach, T., & Carbon, C. C. (2010). Cognitive fluency: High-level processing dynamics in art appreciation. *Psychology of Aesthetics Creativity and the Arts, 4*(4), 214-222. doi:10.1037/a0019648

Boccia, M., Nemmi, F., Tizzani, E., Guariglia, C., Ferlazzo, F., Galati, G., & Giannini, A. M. (2015). Do you like arcimboldo's - esthetic appreciation modulates brain activity in solving perceptual ambiguity. *Behavioural Brain Research, 278*, 147-154. doi:10.1016/j.bbr.2014.09.041

Cupchik, G. C., Vartanian, O., Crawley, A., & Mikulis, D. J. (2009). Viewing artworks: Contributions of cognitive control and perceptual facilitation to aesthetic experience. *Brain and Cognition, 70*(1), 84-91. doi:10.1016/j.bandc.2009.01.003

Gold, B. P., Frank, M. J., Bogert, B., & Brattico, E. (2013). Pleasurable music affects reinforcement learning according to the listener. *Frontiers in Psychology, 4*, 541. doi:10.3389/fpsyg.2013.00541

Hager, M., Hagemann, D., Danner, D., & Schankin, A. (2012). Assessing aesthetic appreciation of visual artworks - the construction of the art reception survey (ARS). *Psychology of Aesthetics Creativity and the Arts, 6*(4), 320-333. doi:10.1037/a0028776

Kreplin, U., & Fairclough, S. H. (2013). Activation of the rostromedial prefrontal cortex during the experience of positive emotion in the context of esthetic experience. an fNIRS study. *Frontiers in Human Neuroscience, 7*(DEC) doi:10.3389/fnhum.2013.00879

Kron, A., Pilkiw, M., Goldstein, A., Lee, D. H., Gardhouse, K., & Anderson, A. K. (2014). Spending one’s time: The hedonic principle in *ad libitum* viewing of pictures. *Emotion, 14*(6), 1087-1101. doi:10.1037/a0037696

Lacey, S., Hagtvedt, H., Patrick, V. M., Anderson, A., Stilla, R., Deshpande, G., . . . Sathian, K. (2011). Art for reward's sake: Visual art recruits the ventral striatum. *Neuroimage, 55*(1), 420-433. doi:10.1016/j.neuroimage.2010.11.027

Markovic, S. (2010). Aesthetic experience and the emotional content of paintings. *Psihologija, 43*(1), 47-64. doi:10.2298/PSI1001047M

Takahashi, S. (1995). Aesthetic properties of pictorial perception. *Psychological Review, 102*(4), 671-683. doi:10.1037/0033-295X.102.4.671

Vessel, E. A., Starr, G. G., & Rubin, N. (2012). The brain on art: Intense aesthetic experience activates the default mode network. *Frontiers in Human Neuroscience, 6*, 1-17. doi:10.3389/fnhum.2012.00066
