# Supplementary material for: Constituents of Music and Visual-Art Related Pleasure – A Critical Integrative Literature Review
Source: Front Psychol. 2017 Jul 20;8:1218. doi: 10.3389/fpsyg.2017.01218 (PMC5517436; doi:10.3389/fpsyg.2017.01218)
Supplement: Supplementary file 2 [file Data_Sheet_2.PDF]

Appendix II

Table 1. A summary of the tabulated data about the reviewed papers.

			KW Role		Methods					Question type				Population			
Authors	Title	Year	Direct	Indirect	Neuroimaging	Questionnaire, Interview	Physiological Measures	Behavioral task	Main Variables	External	Internal	Impact	Journal	N	Women	Men	Mean Age
Scott Parker, Jesse Bascom, Brian Rabinovitz Debra Zellner	Positive and negative hedonic contrast with musical stimuli	2008	X					X	valence, arousal		X		Psychology of Aesthetics, Creativity and the Arts (APA)	32	X	X	Undergrad. Students
Liila Taruffi, Stefan Koelsch	The paradox of music-evoked sadness: An online survey	2014		X		X			7 sections: (1) Core Details; (2) Musical Training and Musical Engagement; (3) Sad Music; (4) Principles Underlying the Evocation of Sadness by Music; (5) Rewarding Aspects of Music-Evoked Sadness; (6) Favourite Sad Music; and (7) Personality Questionnaires. Likert scales + open answers.	X	X		Public Library of Science (PLOS ONE)	772	495	277	28,4
Ernest Mas-Herrero, Robert Zatorre, Antoni Rodriguez-Fornells Josep Marco-Pallarés	Dissociation between musical and monetary reward Responses in specific musical anhedonia.	2014	X				X	X	Physiological measures of emotional arousal;SCR and HR. Pleasure to music: 1-4, chill + overall pleasure: 1-10.Number & the intensity of chills :1-5 + familiarity rating. Monetary task: Magnitude (V2 or V0.2) and valence (gain or loss) of the potential outcome was indicated by a cue at the beginning of each trial. Pleasure experienced with different rewards (food, sex, music, money, exercise, and drugs) using a visual analog scale. In the follow up: we tested differences among groups in music emotion recognition absence or presence of four emotion domains (happy, sad, scary, and peaceful).		X		Current Biology	30	17	13	23
Ernest Mas-Herrero, Josep Marco-Pallares, Urbano Lorenzo-Seva, Robert Zatorre, Antoni Rodriguez-Fornells	Individual differences in music reward experiences	2013	X			X			112 items: music seeking activ-ities, mood regulation, emotion evocation, sensory-motor behavior, social rewarding experiences, and musical memory, social desirability, BIS/BAS and other personality questionnaires. BIS/BAS and physical anhedonia scale.		X		Music Perception	1661	1001	660	28
Benjamin Gold, Michael Frank, Brigitte Bogert, Elvira Brattico	Pleasurable music affects reinforcement learning according to the listener.	2013	X			X		X	Musical background, typical listening patterns (HIMAB). Home listetning task: familiarity, pleasantness, and arousal: Likert scales. Reaction times, and wrong / right (accuracy).			X	Frontiers in Psychology	90	57	33	28
Sandra Garrido, Emery Schubert	Individual differences in the enjoyment of negative emotion in music: A literature review and experiment	2010	X			X			93 items on a Likert scale: Background information, Music empathy, Fantasy Proness, Rumination, Dissociation, Exploratory variables, Empathic concern, Absorbtion		X		Music Perception	59	26	33	22
Valorie Salimpoor, Mitchel Benovoy, Kevin Larcher, Alain Dahger, Robert Zatorre	Anatomically distinct dopamine release during anticipation and experience of peak emotion to music	2011	X		X		X		Chills, dopamine release, emotional arousal: psychophysiological measurements, BOLD, Self-reports: number & intensity of chills, and degree of experienced pleasure.		X		Nature Neuroscience	10	5	5	21

			KW Role		Methods					Question type				Population			
Authors	Title	Year	Direct	Indirect	Neuroimaging	Questionnaire, Interview	Physiological Measures	Behavioral task	Main Variables	External	Internal	Impact	Journal	N	Women	Men	Mean Age
Jonna Vuoskoski, William Thompson, Doris McIlwain, Tuomas Eerola	Who nejoys listening to sad music and why?	2011	X			X		X	3 sets of emotional rating scales representing different theories of emotion. Personality traits: Openness to Experience, empathy.	X	X		Music Perception	148	114	34	24
C. Montag, M. Reuter, N. Axmacher	How one´s favourite song activates the reward circuitry of the brain: Personality matters!	2011	X		X	X			music rated as likeable/unlikeable while choosing it. Personality assessment: Temperament and Character Inventory (TCI).	X	X		Behavioural Brain Research	33	27	6	24
Valorie Salimpoor, Iris van den Bosch, Natasa Kovacevic, Anthony Randal McIntosh, Alain Dagher, Robert Zatorre	Interactions between the nucleus accumbens and auditory cortices predict music reward value	2013	X		X			X	Willingenss to purchase music		X		Science	19	10	9	X
V. Menon, D.J. Levitin	The rewards of music listening: Response and physiological connectivtiy of the mesolimbic system	2005	X		X			X	Rating from not at all pleasant to very pleasant.	X			NeuroImage	13	7	6	19-24
Leonid Perlovsky, Arnaud Cabanc, Marie-Claude Bonniot-Cabanc, Michel Cabanc	Mozart effect, cognitive dissonance, and the pleasure of music	2013	X					X	Multiple choise trainging test + some other qustions. Pleasantness of the music excerpts, answering time.			X	Behavioural Brain Research	64	X	X	14-15
Valorie Salimpoor, Mitchel Benovoy, Gregory Longo, Jeremy Cooperstock, Robert Zatorre	The rewarding aspects of music listening are realted to degree of emotional arousal	2009	X				X	X	Continuous recording of subjective pleasure and sympathetic nervous system activity, an objective measure of emotional arousal, chills.		X		Public Library of Science (PLoS ONE)	32	17	15	22
Marcel Zentner, Didier Grandjean, Klaus Scherer	Emotions evoked by the sound of music: Characterization, classification, and measurement	2008		X		X			515 emotional terms were rated accroding to if the were suitable to describe emotions in general, yes/no. Which of the previous terms would be actually relevent in music?	X			Emotion - American Psychological Association	1393	X	X	X
Anne Blood, Robert Zatorre	Intensely pleasurable responses to music correlate with acitivty in brain regions implicated in reward and emotion	2001	X		X		X	X	Chills	X			Proceedings of the National Academy of Sciences of the United States of America (PNAS)	10	5	5	20-30
Iris van den Bosch, Valorie Salimpoor, Robert Zatorre	Familiarity mediates the relationship between emotional arousal and pleasure during music listening.	2013	X			X	X		Physiological measure (EDA), self report on pleasure, faimiliarity and arousal, questionnaires on musical experience etc.		X		Frontiers in Human Neuroscience	60	32	28	25
Steven Brown, Michael Martinez, Lawrence Parsons	Passive music listening spontaneously engages limbic and paralimbic systems	2004	X		X			X	Subjective ratings of liking and arousal/energy.	X			NeuroReport	10	5	5	34

			KW Role		Methods					Question type				Population			
Authors	Title	Year	Direct	Indirect	Neuroimaging	Questionnaire, Interview	Physiological Measures	Behavioral task	Main Variables	External	Internal	Impact	Journal	N	Women	Men	Mean Age
Heather Chapin, Kelly Jantzen, Scott Kelso, Fred Steinberg, Edward Large	Dynamic emotion and neural responses to music depend on performance expression and listener experience.	2010		X	X			X	Arousal+valence + behavioral task for real time reporting.	X			Public Library of Science (PLoS ONE)	21	14	7	18
Laurette Dubé, Sylvie Morin	Background music plesure and store evaluation intensity effects and psycholgocal mechanisms.	2001	X			X			Questionnaire: unpleasant to pleasant; bad to good. Attitudes towards: sales personel, servicesacpe, store, perceived tempo of music.			X	Journal of Business Research	110	85	25	23
Audrey Laplante, Stephen Downie	The utilitarian and hedonic outcomes of music information-seeking in everyday life	2011	X			X			Questions on: music taste and the place music occupies in their lives. To recall the last music artist or genre they had discovered and liked, and then to try to recall how it happened.Questions regarding the music information sources they used and how they interacted with them. More specifically to talk about the outcomes—hedonic and utilitarian—that contributed to making an interaction with a music IR system satisfying or not when seeking music for leisure purposes. Background information.			X	Library & Information Science Research	15	5	10	24
Marieke Hager, Dirk Hagemann Daniel Danner, Andrea Schankin	Assessing aesthetic Appreciation of visual Artworks - The construction of the art reception survey (ARS)	2012		X		X		X	For the questionnaire contruction: Rating of the endorsement on a five-point rating scale. Assesment of art activity, art attitude, and art knowledge with a general questionnaire. Validation: Raiting of IAPS pictures. The following dimension were also assessed: art activity, art attitude, and art knowledge.	X	X		Psychology of Aesthetics, Creativity and the Arts	224	178	46	28
M. Boccia, F. Nemmi, E. Tizzani, C. Guariglia, F. Ferlazzo, G. Galati, A.M. Giannini	Do you like Archimboldo´s? Esthetic appreciation modulates brain activity in solving perceptual ambiguity	2014		X	X			X	Aesthetic judgement (like / dislike) or calssification task (artistic / non artistic)		X		Behvavioural Brain Research	20	9	11	25
Gerald Cupchik, Oshin Vartanian, Adrian Crawley, David Mikulis	Viewing artworks: Contributions of cognitive control and percpetual facilitation to aeshtetic experience.	2009		X	X			X	Object-identification and aesthetic viewing orientations (pragmatic and aeshtetic vieweing orientation). + 7-point Likert scale (evoking emotions) in the behavioral part + reaction time.	X	X		Brain and Cognition	16	8	8	X
Richard Jacobs, Remco Renken, Frans Cornelissen	Neural correlated visual aesthetics - Beauty as the coalescense of stimulus and internal state.	2012		X	X			X	Judgement of textures for their beauty, naturalness and roughness.	X	X		Public Library of Science (PLoS ONE)	18	8	10	20-39

			KW Role		Methods					Question type				Population			
Authors	Title	Year	Direct	Indirect	Neuroimaging	Questionnaire, Interview	Physiological Measures	Behavioral task	Main Variables	External	Internal	Impact	Journal	N	Women	Men	Mean Age
Shigeko Takahashi	Aeshtetic properties of pictorial perception	1995		X				X	Own expression / intuition? To match drawings with emotions.<br>To match non representational lines drawings with emotion labels on a 7 point scale.<br>The particiapnts had to depict an emotion (no actual measure was used).		X		Psychological Review (American Psychologcial Association)	557	X	X	X
Ute Kreplin, Stephen Fairclough	Activation of the rostromedial prefornal cortex during the experience of positive emotion in the context of esthetic experience. An fNIRS study	2013		X	X	X			During picture viewing ratings for valence and complexity.<br>Pen&paper answers on own, introspective feelings.	X	X		Frontiers in Human Neuroscience	30	15	15	22
Benno Belke, Helmut Leder, Claus Chrstian Carbon, Tilo Strobach	Cognitive Fluency: High-level processing dynamics in art appreciation	2010		X				X	Liking ratings of the paintings on a 4-point Likert scale.	X	X		Psychology of Aesthetics, Creativity and the Arts	20	17	3	26
Simon Lacey, Henrik Hagvedt, Vanessa Patrick, Amy Anderson, Randall Stilla, Gopikrishna Deshpande, Xiaoping Hu, Jao Sato, Srinivas Reddy, K. Sathian	Art for reward´s sake: Visual art recruits the ventralstriatum	2011	X		X			X	Behavioral variables: response time, familiarity ratings and ratings of esthetic preference	X			NeuroImage	8	4	4	23
Slobodan Markovic	Aesthetic experience and the emotional content of paintings	2010		X				X	The developed instrument:<br>unipolar seven-point scales ratings on the chosen descriptors.		X		Psihologija (Serbian Psychological Association)	31	18	13	Undergrad. Students
Assaf Kron, Ariel Goldstein, Maryna Pilkiw, Daniel Lee, Katherine Gardhouse, Adam Anderson	Spending one´s time: The hedonic principle in ad libitum viewing of picures	2014	X				X	X	Affect rating scales (arousal and bipolar valence),<br>complexity scale. Bipolar valence and arousal, EDA and facial EMG activity.	X	X		Emotion (American Psychological Association)	194	118	76	X
Edward Vessel, Gabrielle Starr, Nava Rubin	The brain on art: Intense aesthetic experience activates the default mode network	2012		X	X	X			Nine-item question-naire: evaluative and emotional components of their aesthetic experience. A scale of 1–4 to answer the question "how strongly does this painting move you?" + As a PC questionnaire: Observers were asked to rate the intensity with which each artwork evoked the following evaluative/emotional responses:joy, pleasure, sadness, confusion, awe, fear, disgust, beauty, and thes sublime with a 7-point scale. Prior to fMRI PANAS.		X		Frontiers in Human Neuroscience	16	5	11	28
